# Study on Tumor Budding and Immunoexpression of Cancer Stem Cell Marker ALDH1 in Oral Squamous Cell Carcinoma

**DOI:** 10.7759/cureus.83067

**Published:** 2025-04-27

**Authors:** Siddhartha Tyagi, Smita Chandra, Sampan S Bist

**Affiliations:** 1 Department of Pathology, Himalayan Institute of Medical Sciences, Swami Rama Himalayan University (SRHU), Dehradun, IND; 2 Department of Otorhinolaryngology, Himalayan Institute of Medical Sciences, Swami Rama Himalayan University (SRHU), Dehradun, IND

**Keywords:** aldh1, cancer stem cell, oral squamous cell carcinoma, prognosis, tumor budding

## Abstract

Background

Tumor budding refers to the presence of individual cancer cells or small clusters of cells that break away from the main tumor and are seen at the edge where the tumor is invading surrounding tissue. Aldehyde dehydrogenase 1 is a cancer stem cell marker that causes oxidation of retinol to retinoic acid in early stem cell differentiation. This study aimed to examine tumor budding in oral squamous cell carcinoma and explore how it relates to various clinical and pathological factors. A study of cancer stem cell marker ALDH1 was also done with correlation to tumor budding and prognostic markers.

Material and methods

A prospective study was carried out in a pathology department over one year and included all newly diagnosed cases of oral squamous cell carcinoma that had undergone surgical resection. Both the gross specimens and the tissue sections stained with hematoxylin and eosin (H&E) from paraffin-embedded samples were examined for tumor size, histopathological grade, in situ carcinoma, necrosis, lymphovascular emboli, perineurial invasion, and lymph node metastasis. Tumor budding was evaluated for every case by observing tumor buds (clusters of a single tumor cell or a small cluster of tumor cells (<5) at the invasive front of the tumor) at 40x. Immunohistochemical analysis for ALDH-1 was performed on paraffin-embedded histopathological sections.

Results

A total of 50 cases were included in the study, with a mean age of 49 years and a male-to-female ratio of 7.3:1. The most common site of carcinoma was buccal mucosa (34%), followed by tongue (30%). Ninety percent (90%) of cases were moderately differentiated squamous cell carcinoma, with 82% of cases showing lymphovascular emboli and 32% showing perineural invasion. The highest number of cases, 22% (11), were classified as pT4aN0Mx, followed by 16% (8 cases each) classified as pT2N0Mx and pT3N0Mx. Sixty-six percent (66%; n=33) of cases showed high-grade tumor budding while 34% (n=17) showed low-grade tumor budding. Eighty-eight point eight percent (88.8%; n=32) of cases with high-grade tumor budding as well as lymphovascular invasion were observed with a statistically significant association (p=0.042). The study found a statistically significant link between high-grade tumor budding and the presence of lymph node metastasis (p=0.003). Fifty-eight percent (58%) of cases showed positive immunostaining of ALDH1 with varying intensity from weak to strongly positive. ALDH1 expression showed a significant association with high-grade tumor budding, with the result being statistically highly significant (p = 0.0001).

Conclusion

High-grade tumor budding is associated with increased expression of cancer stem cell marker ALDH1 in oral squamous cell carcinoma. It is also associated with lymphovascular invasion and lymph node metastasis, suggesting a poor prognosis in these patients. It is recommended that tumor budding should be included in histopathological reports of oral carcinoma. ALDH1 may be explored as a potential target for therapy in oral carcinoma. The authors suggest that further larger studies with extended follow-up may be done to establish the role of tumor budding and ALDH1 in the prognosis of oral carcinoma.

## Introduction

According to Global Cancer Observatory: CANCER TODAY (GLOBOCAN) 2022 and WHO, the age-standardized incidence rate (ASR) of oral cavity cancer worldwide is 4 per 100,000 when observed in both sexes. However, in India, the incidence of oral cavity carcinoma is higher compared to the world (9.9/10,00,000) with a mortality of 5.6/10,00,000 [1]. Tumor budding refers to the presence of individual cancer cells or small clusters of cells that break away from the main tumor and are seen at the edge where the tumor is invading surrounding tissue [2]. It has been investigated in various types of cancer, and the International Tumor Budding Consensus Conference (ITBCC) has established a standardized scoring system specifically for reporting tumor budding in gastrointestinal cancer [3]. In oral carcinoma, it is also emerging as an important prognostic marker, and it is being suggested that this should be included in reports. Aldehyde dehydrogenase 1 (ALDH1), a cytosolic enzyme, is a cancer stem cell marker that causes oxidation of retinol to retinoic acid in early stem cell differentiation [4]. It is also considered an indicator of epithelial-mesenchymal transition, which plays a role in metastasis. In addition, studies are ongoing to study the role of cancer stem cells in tumor heterogeneity and treatment response [5,6].

This study was undertaken to evaluate tumor budding in oral squamous cell carcinoma (OSCC) and to analyze its correlation with various clinical and pathological features. In addition, the objective of the present study was to correlate the expression of cancer stem cell marker ALDH1 with tumor budding and prognostic markers in OSCC. This would be further helpful in the evaluation of this cancer stem cell marker in targeted therapy of OSCC.

## Materials and methods

**Figure 1:**
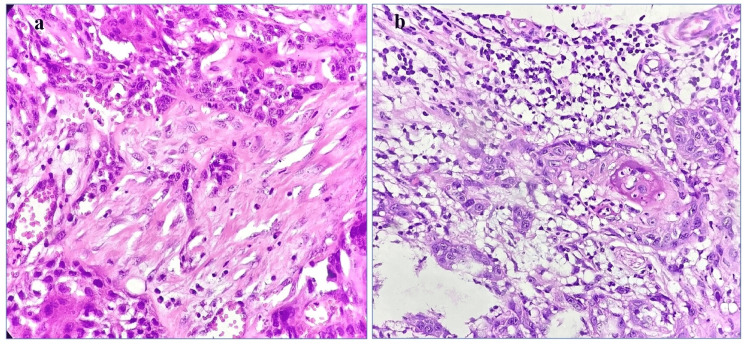
a) Histopathology section showing low-grade tumor budding in oral squamous cell carcinoma (HE;x 40); b) Histopathology section showing high-grade tumor budding in oral squamous cell carcinoma (HE;x 40)

This prospective study was carried out in the pathology department of Himalayan Institute of Medical Sciences, Swami Rama Himalayan University (SRHU), Dehradun, India, over one year and included all newly diagnosed cases of OSCC that had undergone surgical resection. Small biopsies or cases that had received any prior treatment (radiation or chemotherapy) were excluded from the study. For each case, important clinical information, imaging results, and laboratory test findings were recorded. Both the gross specimens and the tissue sections stained with hematoxylin and eosin (H&E) from paraffin-embedded samples were examined for tumor size, histopathological grade, in situ carcinoma, necrosis, lymphovascular emboli, perineurial invasion, and lymph node metastasis. Tumor budding was evaluated for every case by observing tumor buds (clusters of a single tumor cell or a small cluster of tumor cells (<5) at the invasive front of the tumor) at 40x. Low-grade tumor budding was <20 buds per 10 high-power field, and high-grade tumor budding was > or = 20 buds per 10 high-power field (Figures 1a-1b).

**Figure 2:**
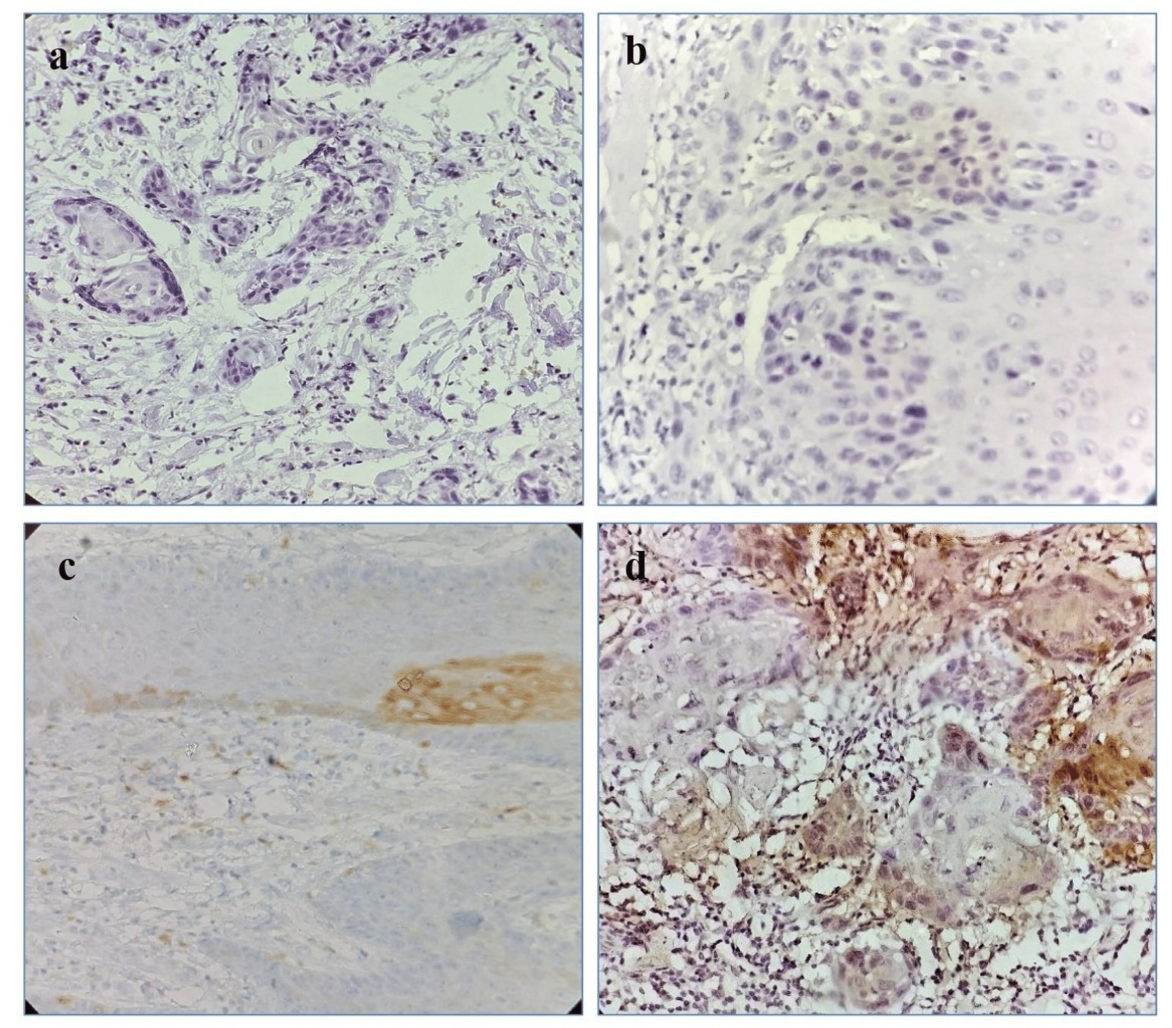
ALDHI immunoexpression showing a) negative expression, b) weak expression, c) moderate expression, and d) strongly positive expression in oral squamous cell carcinoma (ALDH1 immunostaining; x40) ALDH1: aldehyde dehydrogenase 1

An immunohistochemical analysis for ALDH1 was performed on paraffin-embedded histopathological sections. Deparaffinization of sections was done, and antigen was retrieved by the microwave technique (at high power). It was cooled for 5-10 minutes and washed in buffer solution for 5-10 minutes. Blocking of endogenous enzyme activity was done by placing the sections in a Koplin jar filled with 0.3% H2O2 (1 ml of 30% H2O2 + 9 ml methanol) for 10-13 minutes. After the incubation, the slides were taken out and washed in phosphate-buffered saline (PBS) for five minutes. After blocking endogenous peroxidase activity with a power block for 10-15 minutes, the excess was wiped off. The slides were then incubated with a primary mouse polyclonal antibody to ALDH1 for 2 hours at 25-30 °C in a moist chamber. Excess antibody was wiped off, and the slide was placed in a buffer for five minutes. It was incubated with a super-enhancer and kept for 30 minutes at 25-30 °C in a moist chamber. It was incubated with horse-radish peroxidase (HRP) polymer in a moist chamber for 30 minutes at 20-25 °C temperature. The excess polymer was wiped off and placed in a buffer for five minutes. It was incubated with peroxidase substrate solution 33’ diaminobenzidine tetrahydrochloride (DAB/AEC) 1 ml, and 38-40 microliter DAB chromogen was added for 10-15 minutes at 25-30 °C temperature in a moist chamber. It was rinsed with distilled water and counterstained with hematoxylin in just one dip, followed by one dip in 1% acid alcohol before washing in running tap water for 10-15 minutes. Dehydration was done in alcohol, upgrading from 70%, 80%, 90%, and then absolute alcohol. Drying and mounting were done with DPX. Allred scoring of ALDH1 immunoexpression was done by considering the percentage of positive cells and the intensity of staining of tumor bud cells. The percentage positivity of ALDH1 was reported as an Allred score of 0 when no cell was positive for immunostaining, score 1 for <1%, score 2 for 1-10%), score 3 for 11-33%), score 4 for 34-66%), and score 5 for 67-100% positive cells. The intensity of immunostain was also graded as negative, weak, moderate, and strongly positive (Figures 2a-2d).

The final ALDH1 scoring was done as negative, low, and high by combining the percentage of positive cells and the intensity of scoring.

## Results

The study comprised a total of 50 cases of OSCC with a male-to-female ratio of 7.3:1. Ninety-eight percent (98%; n=49) of cases were associated with a history of tobacco chewing and smoking. Table 1 shows the distribution of cases according to age group. It was observed that the maximum number of cases (36%) was in the age group 41-50 years. The mean age was 49 years, and the range was 30-71 years.

**Table 1:** Distribution of cases with the age group (n=50)

Age Group	Frequency	Percentage (%)
30 – 40	11	22
41 – 50	18	36
51 – 60	14	28
> 60	7	14
Total	50	100

Table 2 shows the presenting symptoms of cases, and it was observed that all the cases had a history of pain, and 36% (n=18) had difficulty chewing food.

**Table 2:** Distribution of cases according to presenting symptoms

Symptoms	Frequency	Percentage (%)
Pain	50	100
Difficulty in chewing	18	36.0
Irritation	6	12.0
Difficulty in opening mouth	4	8.0
Difficulty in speaking	1	2.0
Itching	2	4.0
Swelling	4	8.0

The most common site of carcinoma was the buccal mucosa (34%), followed by the tongue (30%), and 56% of cases involved the left side of the oral cavity. The tumor size was 2-5 cm in the maximum number of cases (78%), and the depth of invasion was 0-0.5 cm in 50% of cases. Ninety percent (90%) of cases were of moderately differentiated squamous cell carcinoma, with 82% of cases showing lymphovascular emboli and 32% showing perineural invasion. According to pathological tumor-node-metastasis (pTNM) staging, the highest number of cases, 22% (11), were classified as pT4aN0Mx, followed by 16% (8 cases each) classified as pT2N0Mx and pT3N0Mx. Ninety-six percent (96%) of the patients were still alive six months after being diagnosed. Sixty-six percent (66%; n=33) of cases showed high-grade tumor budding while 34% (n=17) of cases showed low-grade tumor budding. Eighty-eight point eight percent (88.8%; n=32) of cases showing high-grade tumor budding also exhibited lymphovascular invasion, and this association was statistically significant (p = 0.042). A significant association was found between high-grade tumor budding and lymph node metastasis, with the result being statistically meaningful (p = 0.003). Table 3 presents how the cases are distributed according to the intensity scores of ALDH1 immunostaining. It shows that 58% of cases showed positive staining with varying intensity from weak to strongly positive.

**Table 3:** Distribution of cases on the basis of the intensity score of ALDH1 immunostaining (n=50) ALDH1: aldehyde dehydrogenase 1

Allred Scoring (Intensity Score)			
Negative	Weak	Moderate	Strongly Positive
21 (42%)	14 (28%)	07 (14%)	08 (16%)

Table 4 shows the distribution of cases on the basis of the percentage of cells showing positivity of ALDH1 immunostaining.

**Table 4:** Distribution of cases on the basis of the percentage score of tumor buds positive for ALDH1 (n=50) ALDH1: aldehyde dehydrogenase 1

Allred Scoring (Percentage Score)					
0 (0%)	1 (<1%)	2 (1-10%)	3 (11-33%)	4 (34-66%)	5 (67-100)
21 (42%)	06 (12%)	13 (26%)	08 (16%)	02 (4%)	00 (0%)

Table 5 illustrates the relationship between tumor budding and ALDH1 expression as determined by immunohistochemistry in cases of OSCC. It indicates that positive ALDH1 expression was significantly linked to high-grade tumor budding with a strong statistical significance (p = 0.0001). 

**Table 5:** Association between tumor budding and immunohistochemistry of ALDH1 in oral squamous cell carcinoma ALDH1: aldehyde dehydrogenase 1

Immunohistochemistry		Tumor Budding			P value
		Low grade	High grade	Total	
ALDH1	Positive	1 (5.9%)	28 (84.8%)	29 (58%)	0.0001 (χ2≈10.8280
	Negative	16 (94.1%)	5 (15.2%)	21 (42%)	
	Total	17 (100%)	33 (100%)	50 (100%)	

## Discussion

The crude incidence rate of oral cancer in India is 14.5%, with a mortality of 7.9%, and the most common site being the lip and oral cavity (55.6%) [1]. In the present study, the buccal mucosa and tongue were the most common sites for oral carcinoma. Tobacco and smoking are known risk factors for oral carcinoma, and studies from India and worldwide have observed an increased association of these addictions with oral carcinoma. A recent study from Kerala has concluded that the habit of betel nut chewing, smoking, and alcohol intake was present in 90% of the patients [7]. Anwar et al. have also observed in their study from Pakistan that the odds of developing buccal mucosa tumors in chewers (of any type of substance) and gutka users were two and four times higher than those for non-chewers, respectively [8]. The present study also observed that 98% of cases were associated with tobacco chewing and smoking, and the maximum number of cases was in the buccal mucosa. It was also observed that the maximum cases presented at a higher stage. A likely reason for the delayed diagnosis of oral carcinoma is that patients often ignore precancerous conditions and reside in remote hilly areas of the state with limited access to healthcare. Tumor budding is under investigation as a prognostic indicator in multiple malignancies; however, a standardized scoring system has yet to be established [9,10]. Tumor budding is currently being extensively studied in oral carcinoma to establish its prognostic significance, but no definitive guidelines have been established [11]. Abd Raboh NM et al. have concluded that tumor budding shows high statistically significant relations with site, grade, tumor stage, lymph node stage, extracapsular invasion, and vascular invasion in laryngeal carcinoma [12]. The current study found that high-grade tumor budding had a statistically significant association with both lymphovascular invasion and lymph node metastasis. 

ALDH1 is being recently explored as a marker of cancer stem cells in various malignancies and their prognosis [13,14]. The present study also aimed to evaluate the presence of cancer stem cell markers in tumor buds, which could have potential implications for prognosis in OSCC. There are 19 isoforms of ALDH, and their dysregulation has been associated with different carcinomas. However, it remains unclear whether ALDH1 has a favorable or unfavorable effect on cancer prognosis [15]. In oral carcinoma, high ALDH1 immunoexpression may indicate a poorer prognosis, but several studies have found no significant correlation between ALDH1 expression and clinicopathological features or patient prognosis in oral cancer [16,17].

The present study found that 58% of the cases showed ALDH1 positivity in tumor buds with intensity levels varying from weak to strong, as well as differences in the percentage of positive cells. There was a statistically significant correlation of high-grade tumor budding with the positive immunoexpression of ALDH1. This suggests the ability of tumor buds to exhibit invasive and metastatic properties of cancer stem cells. Although this phenomenon has been studied in colon cancer models previously, its role in oral carcinoma is being explored presently. Marangon Junior H et al. observed that ALDH1 expression was higher in the budding area than in the area outside the budding in cases of OSCC with high-intensity tumor budding [18]. Although they observed no association between ALDH1 expression and tumor budding (p > 0.05), this may be due to the inclusion of incisional biopsy cases in the study. However, to overcome this limitation, the present study excluded all the cases of incisional biopsy.

ALDH1 expression is also being investigated as a source of targeted therapy in various carcinomas. Feng et al. studied the targeting of colorectal cancer with small-molecule inhibitors of ALDH1B1, while Bu et al. targeted ALDH1 in triple-negative breast carcinoma [19,20].

A key limitation of this study was the relatively small sample size and the short duration of patient follow-up. Therefore, further studies with a larger number of cases and extended follow-up should be done to validate the role of ALDH1 in the prognosis and therapy of oral squamous cell carcinoma.

## Conclusions

High-grade tumor budding is associated with an increased expression of cancer stem cell marker ALDH1 in oral squamous cell carcinoma. It is also associated with lymphovascular invasion and lymph node metastasis, suggesting a poor prognosis in these patients. It is recommended that tumor budding should be included in histopathological reports of oral carcinoma. ALDH1 may be explored as a potential target for therapy in oral carcinoma. The authors suggest that further larger studies with extended follow-up may be done to establish the role of tumor budding and ALDH1 in the prognosis of oral carcinoma.
